# Supplementary material for: Dickkopf1 destabilizes atherosclerotic plaques and promotes plaque formation by inducing apoptosis of endothelial cells through activation of ER stress
Source: Cell Death Dis. 2017 Jul 13;8(7):e2917–. doi: 10.1038/cddis.2017.277 (PMC5550842; doi:10.1038/cddis.2017.277)
Supplement: Supplementary Table 1 [file cddis2017277x1.docx]

**Supplement Table 1: Body weight, serum lipid profiles and glucose concentration**

|  | NS | GFP | shDKK1 | DKK1 | *p* |
| --- | --- | --- | --- | --- | --- |
| BW（g） | 23.95±0.78 | 24.72±0.88 | 26.43±0.61 | 25.92±0.95 | ns |
| TC (mmol/L) | 21.68±0.35 | 22.20±0.42 | 23.26±0.39 | 22.73±0.78 | ns |
| TG (mmol/L) | 2.03±0.06 | 1.92±0.05 | 2.06±0.06 | 1.98±0.09 | ns |
| HDL-C (mmol/L) | 5.84±0.09 | 5.36±0.18 | 5.80±0.13 | 5.50±0.17 | ns |
| LDL-C (mmol/L) | 2.64±0.07 | 2.66±0.06 | 2.75±0.08 | 2.60±0.12 | ns |
| glucose (mmol/L) | 5.69±0.41 | 5.38±0.55 | 5.50±0.29 | 5.84±0.41 | ns |

BW: body weight; TC: total cholesterol; TG: triglyceride; HDL-C: high density lipoprotein-cholesterol; LDL-C: low density lipoprotein-cholesterol. Data are mean ±SEM.
