# Supplementary material for: Dickkopf1 destabilizes atherosclerotic plaques and promotes plaque formation by inducing apoptosis of endothelial cells through activation of ER stress
Source: Cell Death Dis. 2017 Jul 13;8(7):e2917–. doi: 10.1038/cddis.2017.277 (PMC5550842; doi:10.1038/cddis.2017.277)
Supplement: Supplementary Table 2 [file cddis2017277x2.docx]

**Supplement Table 2**

| Materials and Reagents |  |
| --- | --- |
| β-actin | Cell Signaling Technology, 3700,WB(diluted1:1000) |
| DKK1 | Abcam,ab109416,WB( diluted 1:500); IHC(diluted1:100) |
| Caspase-3 | Cell Signaling Technology, 9662,WB（diluted 1:300） |
| Cleaved caspase-3 | Abcam,ab32042,WB(diluted 1:500); Cell Signaling Technology, 9662,WB(diluted 1:300);IHC(diluted 1:100) |
| Bax | Abcam,ab32503,WB(diluted 1:1000);IHC(diluted1:200) |
| Bcl-2 | Abcam,ab32124,WB(diluted 1:1000);IHC(diluted1:200), |
| IL-6 | Abcam,ab191194, IHC(diluted1:200); ab6672,IHC(diluted1:400), WB(diluted 1:1000); |
| IL-1β | Abcam, ab9722, IHC(diluted1:100), WB(diluted 1:1000); |
| TNF-α | Abcam, ab6671, IHC(diluted1:100), WB(diluted 1:1000); |
| MCP-1 | Abcam, ab25124, IHC (diluted1:100); ab9669, IHC (diluted1:100) , WB(diluted 1:1000); |
| Bip/GRP78 | Cell Signaling Technology, 3177,WB(diluted 1:1000) |
| ATF6 | Abcam，ab203119，WB(diluted 1：500);IHC(diluted 1:50) |
| IRE1α | Cell Signaling Technology, 3294, WB(diluted 1:1000) |
| p-IRE1α | Abcam,ab124945,S724,WB(diluted 1:1000) |
| XBP1 | Abcam,ab109221 ,WB(diluted1:1000) |
| Capase-12 | Abcam,ab62484,IHC(diluted 1:1000) |
| eif2α | Cell Signaling Technology, 5324,WB(diluted 1:1000); IHC(diluted1:50) |
| p-eif2α | Abcam,ab32157,S51,WB(diluted 1:500);IHC(diluted1:200) |
| CHOP | Abcam,ab179823,WB(diluted 1:1000) |
| p-JNK | Cell Signaling Technology,9251,WB(diluted1:1000) |
| JNK | Cell Signaling Technology,9252, WB(diluted1:1000) |
| MOMA-2 | Abcam,ab33451,IHC(diluted 1:50) |
| α-SMA | Abcam,ab5694,IHC(diluted 1:200) |
| Recombinant human DKK1 (rDKK1) | R&D Systems (Minneapolis, MN, USA)， 3ng/ml |
| 4-PBA | Sigma (St. Louis, MO, USA), dissolved in PBS for 1M for storage |
| Salubrinal | Sigma (St. Louis, MO, USA) , dissolved in dimethyl sulfoxide (DMSO; Sigma) for 100mM for storage |
| LY294002（inhibitor of JNK） | Cell Signaling Technology, 8177 |
| FH535(inhibitor of WNT/β-catenin) | Selleck(Houston , USA) dissolved in dimethyl sulfoxide (DMSO; Sigma) for 200mM for storage |
| IM-12 | Selleck(Houston , USA) dissolved in dimethyl sulfoxide (DMSO; Sigma) for 200mM for storage |
| Norepinephrine | Sigma,A0937 |
| Acetylcholine | Sigma, A6625 |
| HUVEC | ScienCell, Cat.No. 8000 |
| ECM | ScienCell, Cat.No. 1001 |
| ox-LDL | Yiyuan Biotechnologies(Guangzhou, China),150 µg/ml |
| siRNA sequence |  |
| Negative control(5’-3’) | Sense：UUCUCCGAACGUGUCACGUTT |
|  | Antisense：ACGUGACACGUUCGGAGAATT |
| DKK1(5’-3’) | Sense:GCUUCACACUUGUCAGAGATT |
|  | Antisense：UCUCUGACAAGUGUGAAGCCT |
| CHOP(5’-3’) | Sense: GAGCUCUGAUUGACCGAAUTT |
|  | Antisense: AUUCGGUCAAUCAGAGCUCTT |
| IRE1α(5’-3’) | Sense: CUCCGAGCCAUGAGAAAUATT |
|  | Antisense: UAUUUCUCAUGGCUCGGAGTT |
| Primers | **Sequence (5′-3′)** |
| β-actin-RT-F | Forward: CGTGCGTGACATTAAGGAGA |
| β-actin- RT-R | Reverse: CACCTTCACCGTTCCAGTTT |
| DKK1-RT -F | Forward: ATAGCACCTTGGATGGGTATTCC |
| DKK1-RT -R | Reverse: CTGATGACCGGAGACAAACAG |
