# Supplementary figures and images for: Dickkopf1 destabilizes atherosclerotic plaques and promotes plaque formation by inducing apoptosis of endothelial cells through activation of ER stress
Source: Cell Death Dis. 2017 Jul 13;8(7):e2917–. doi: 10.1038/cddis.2017.277 (PMC5550842; doi:10.1038/cddis.2017.277)

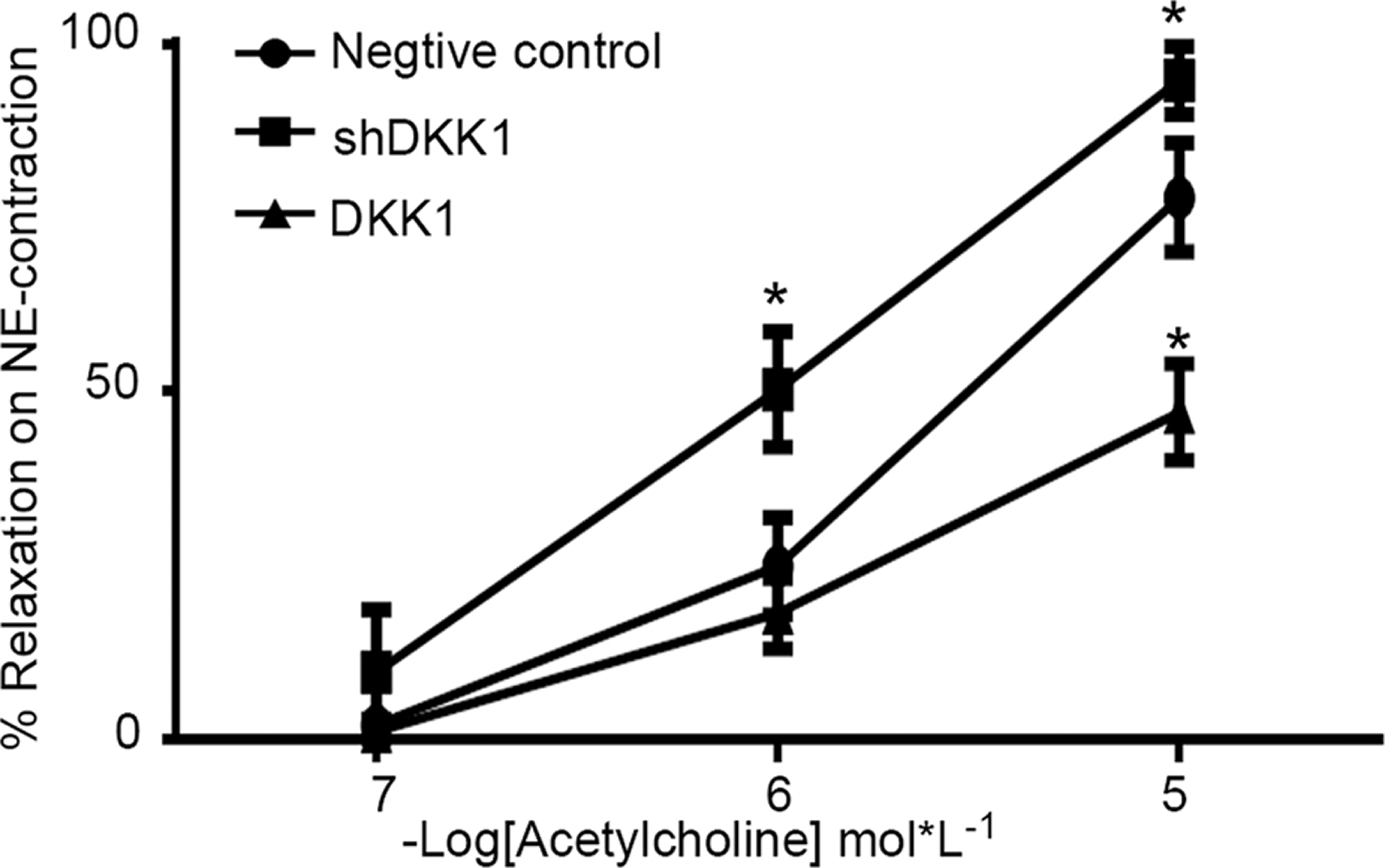

Supplement: Supplementary Figure 1 [file cddis2017277x3.tif]

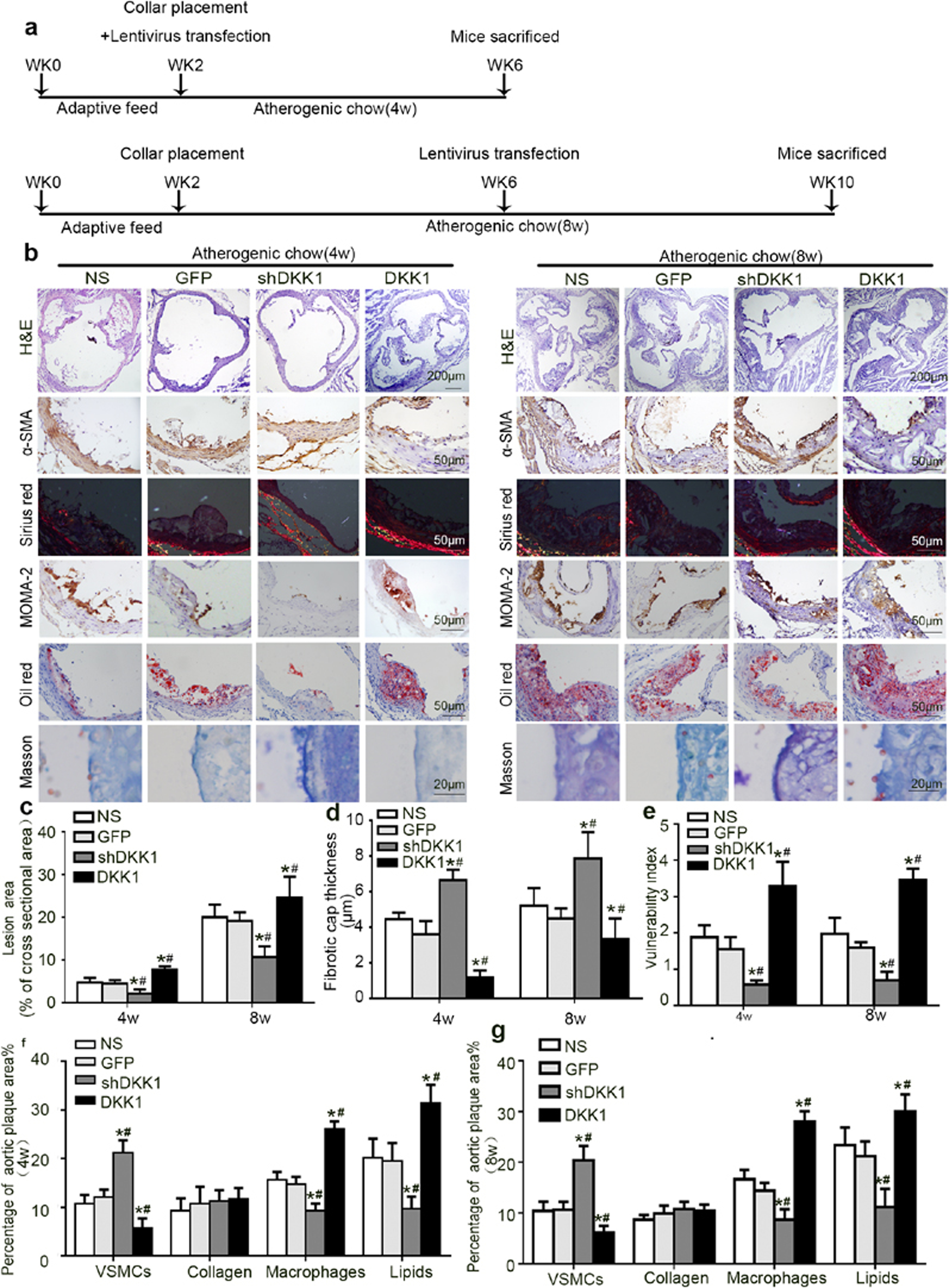

Supplement: Supplementary Figure 2 [file cddis2017277x4.tif]

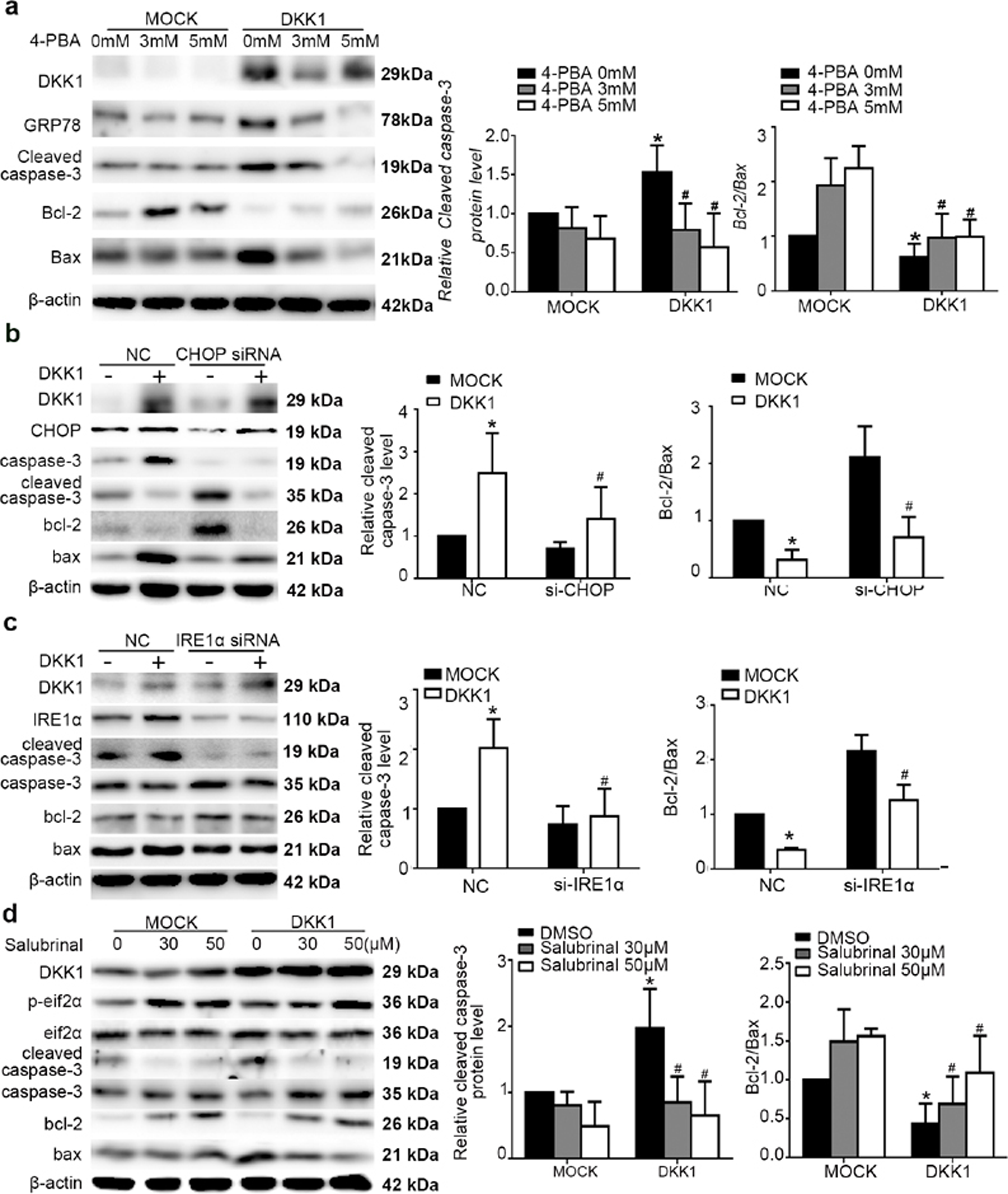

Supplement: Supplementary Figure 3 [file cddis2017277x5.tif]

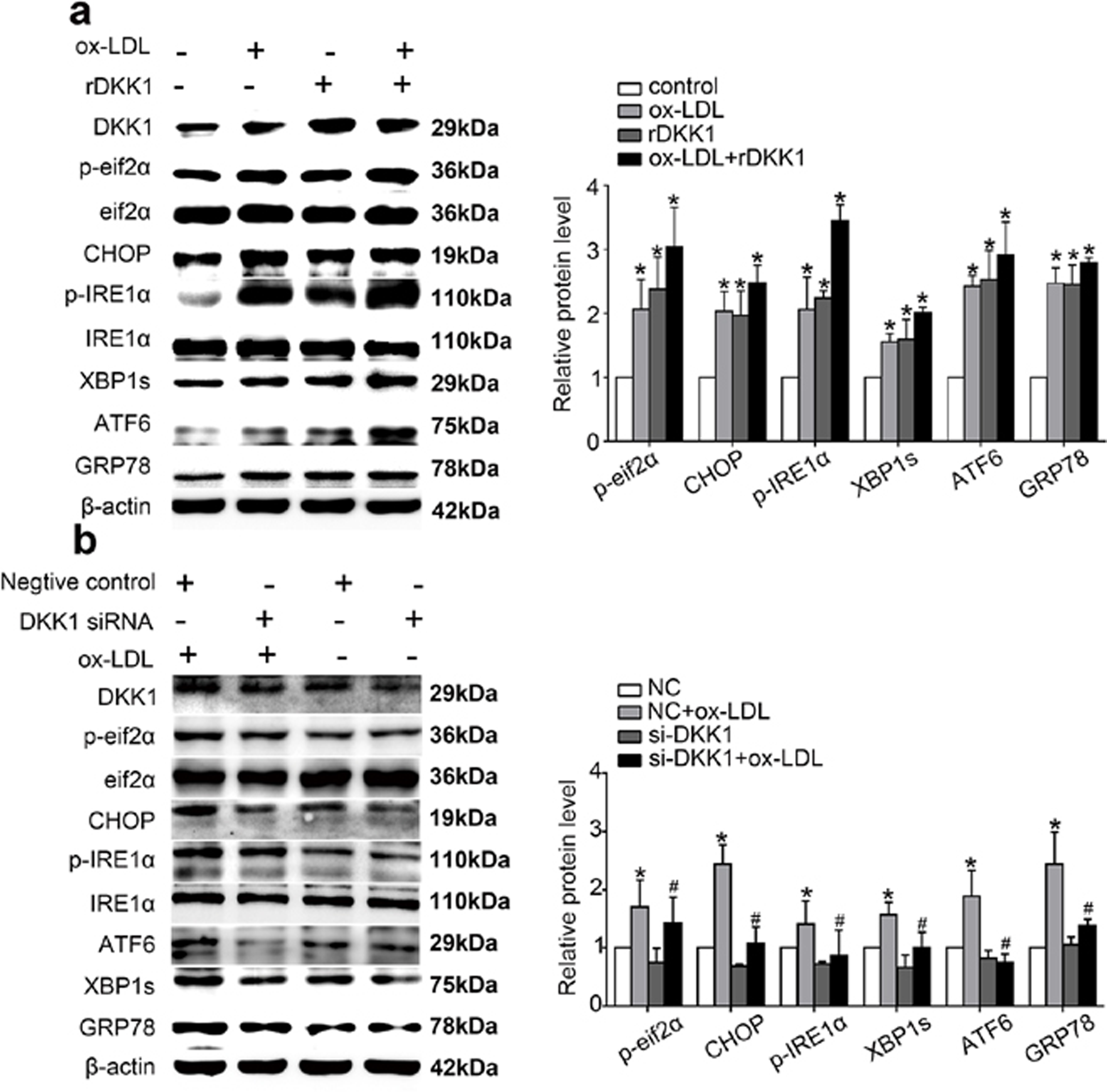

Supplement: Supplementary Figure 4 [file cddis2017277x6.tif]

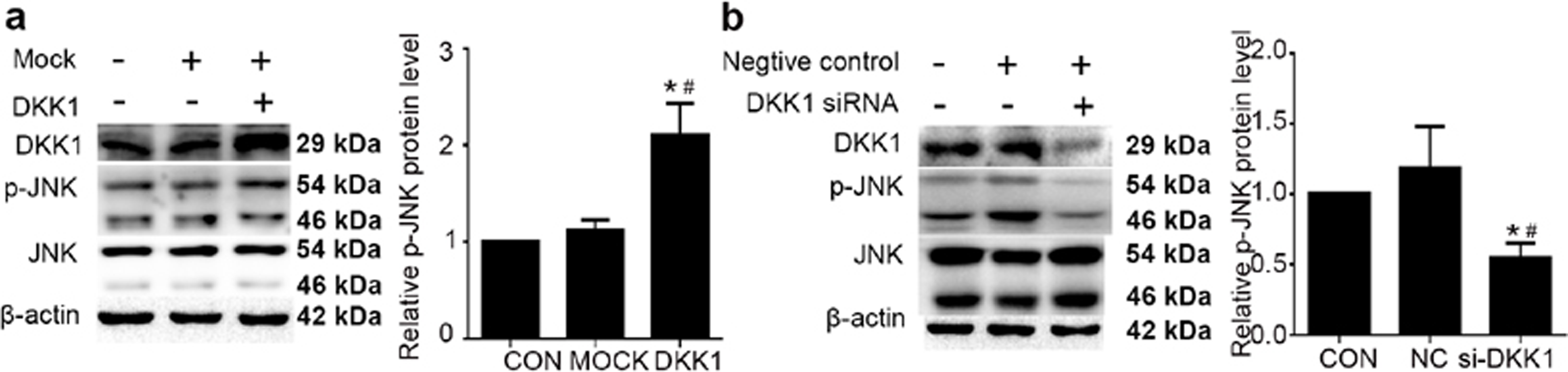

Supplement: Supplementary Figure 5 [file cddis2017277x7.tif]

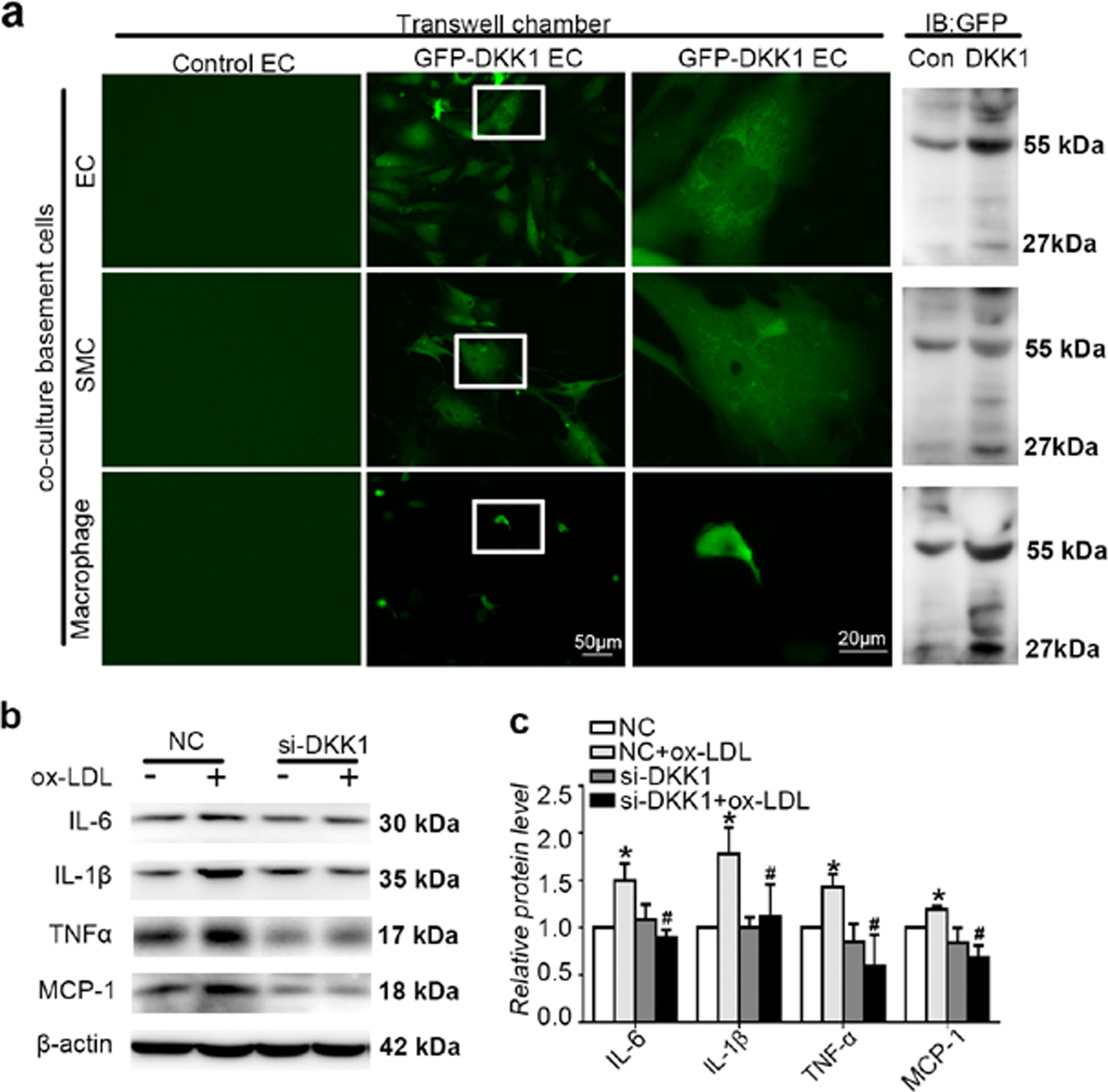

Supplement: Supplementary Figure 6 [file cddis2017277x8.tif]

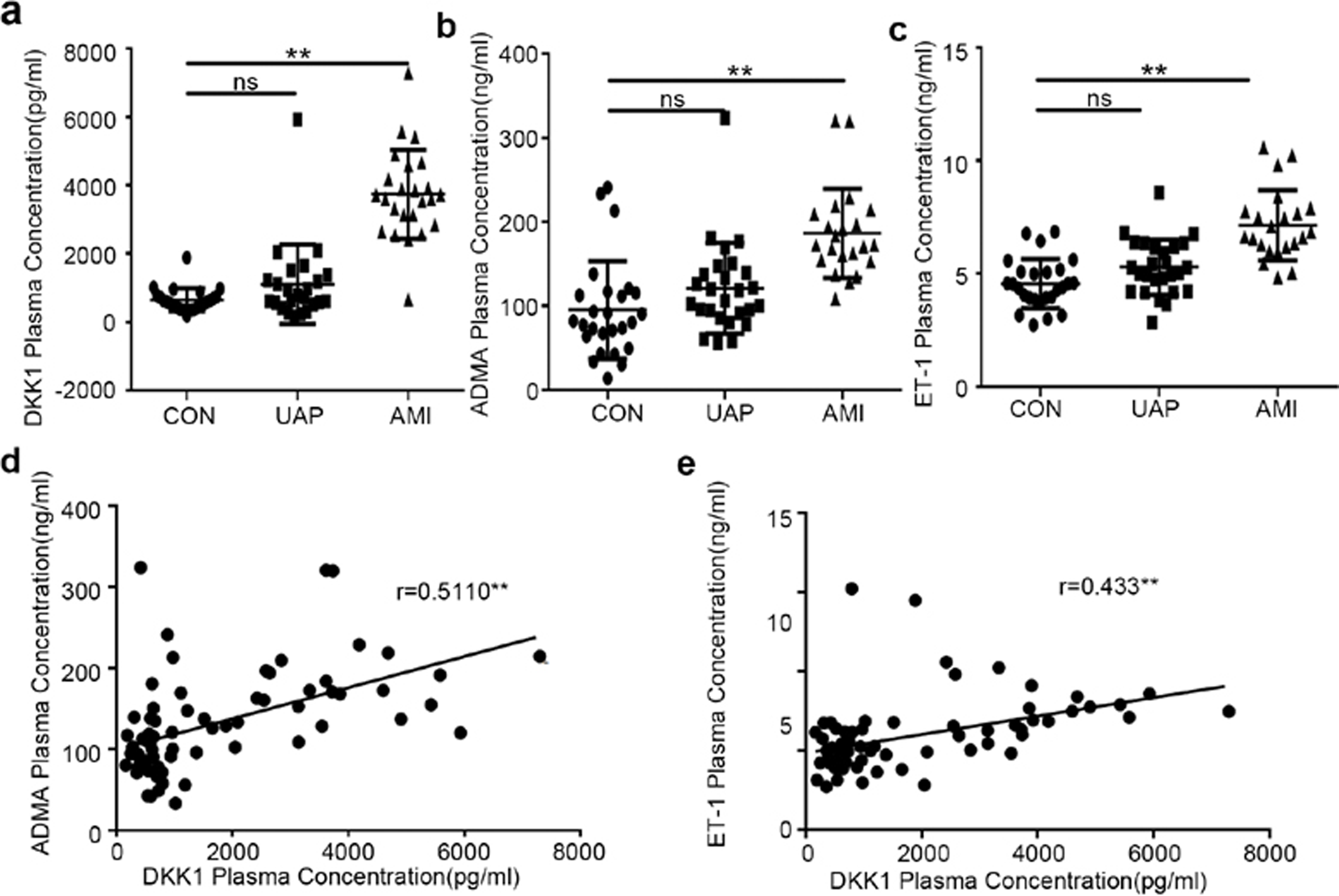

Supplement: Supplementary Figure 7 [file cddis2017277x9.tif]
