# Supplementary material for: Dickkopf1 destabilizes atherosclerotic plaques and promotes plaque formation by inducing apoptosis of endothelial cells through activation of ER stress
Source: Cell Death Dis. 2017 Jul 13;8(7):e2917–. doi: 10.1038/cddis.2017.277 (PMC5550842; doi:10.1038/cddis.2017.277)
Supplement: Supplementary Figure Legends [file cddis2017277x10.doc]

# Dickkopf1 destabilizes atherosclerotic plaques and promotes plaque formation by inducing apoptosis of endothelial cells through activation of ER stress

Mingxue Di, MD^12^*; Lin Wang, MD^123^*; Mengmeng Li,MD^12^ ; Yu Zhang,MD^12^ ; Xinxin Liu,PHD^12^; Renya Zeng^12^; Han Wang^12^ ; Yifei Chen^12^ ; Weijia Chen^12^，Yun Zhang, MD^12^; Mei Zhang, MD^12+^

1 The Key Laboratory of Cardiovascular Remodeling and Function Research, Chinese Ministry of Education and Chinese Ministry of Health, Qilu Hospital, Shandong University, Shandong 250012, P.R. China

2 The State and Shandong Province Joint Key Laboratory of Translational Cardiovascular Medicine, Qilu Hospital, Shandong University, Shandong 250012, P.R. China

3.The second hosipital of Shandong University, Jinan, Shandong 250012, P.R. China

* The two authors contributed equally to this work and should be considered co-first authors.

+ Corresponding author at: Department of Cardiology, Qilu Hospital, Shandong University, No.107, Wen Hua Xi Road, Jinan, Shandong, 250012, China. Tel.: +86 531 82169139; fax: +86 531 86169356; E-mail: [daixh@vip.sina.com](mailto:daixh@vip.sina.com)

Mingxue Di: [dimingxue@163.com](mailto:dimingxue@163.com); Lin Wang: [wanglin54202003@163.com](mailto:wanglin54202003@163.com);

Mengmeng Li: zpyzlmm90128@163.com ; Yu Zhang: [zhangyusdu@sina.cn](mailto:zhangyusdu@sina.cn);

Xinxin Liu: 6 ; Renya Zeng: [lihongqi521@sina.cn](mailto:lihongqi521@sina.cn);

Han Wang: 2310004129@qq.com ; Yifei Chen: [bbishy@gmail.com](mailto:bbishy@gmail.com);

Weijia Chen: [langganc@163.com](mailto:langganc@163.com)； Yun Zhang: [zhangyun@sdu.edu.cn](mailto:zhangyun@sdu.edu.cn)

**Abstract**

Several clinical studies reported that Dickkopf1 (DKK1) plasma levels are correlated with atherosclerosis. However, the impact of DKK1 on the formation and vulnerability of atherosclerotic plaques remains elusive. This study investigated DKK1’s effects on enlargement and destabilization of plaques by targeting endothelial cells and assessing the possible cellular mechanisms involved. The effects of DKK1 on atherogenesis and plaque stability were evaluated in ApoE−/− mice using lentivirus injections to knock down and knock in the DKK1 gene. The presence of DKK1 resulted in enlarged and destabilized atherosclerotic lesions and increased apoptosis, while silencing of DKK1 alleviated plaque formation and vulnerability in the whole progression of atherosclerosis. DKK1 expression was upregulated in response to ox-LDL treatment in a time- and concentration-dependent manner on human umbilical vein endothelial cell (HUVEC). The interference of DKK1 reversed ox-LDL-induced apoptosis in HUVECs. The mechanism underlying this effect was DKK1’s activation of the JNK signal transduction pathway and inhibition of canonical Wnt signaling, following by activation of the IRE1α and eif2α/CHOP pathways. In conclusion, DKK1 promotes plaque formation and vulnerability partly by inducing apoptosis in endothelial cells, which partly through inducing the JNK-endoplasmic reticulum stress pathway and inhibiting canonical Wnt signaling.

**Introduction**

Recent studies have found that acute coronary syndrome (ACS) is associated with both the sudden rupture of atherosclerotic plaques and the rapid development of these plaques. While atherogenesis remains incompletely understood, studies of atherogenesis pathology suggest that multiple cellular malfunctions, including endothelial cell (EC) dysfunction and vascular integrity disruption, are involved, as well as increases in inflammatory cell numbers, the production of cytokines, the proliferation and migration of vascular smooth muscle cells (VSMCs), the activation of monocytes and macrophages, and neovascularization. Pathological biomechanical and haemodynamic changes (e.g., [oxidative damage](https://www.ncbi.nlm.nih.gov/pubmed/25392110), shear stress) result in these events, which are closely correlated with plaque stability.^1^ EC dysfunction has been observed in atherosclerotic lesions in both humans and animals and eventually leads to apoptosis and the development of atherosclerosis.^2, 3^ Endothelial dysfunction leads to proinflammatory activation, generates autocrine and paracrine signaling loops, and influences other type of cells that are involved in atherogenesis.^4^ EC apoptosis occurs throughout the early stages of atherosclerosis and plays important roles in plaque regression and plaque instability,^5, 6^ which are caused by various factors, particularly by oxidized low-density lipoprotein (ox-LDL).^1, 7^ Thus, inhibition of EC apoptosis may be a useful therapeutic strategy for ameliorating plaque instability.

Dickkopf1 (DKK1), a secretory glycoprotein, can block the Wnt pathway by competitively binding to receptors (e.g., LRP5/6, Kremen) on the cell membrane.^8^ Ueland et al. found that DKK1 expression was stronger in von Willebrand factor (vWF)-positive ECs and in CD68-positive macrophages than in other areas of plaques.^9^ They also found that DKK1 participated in platelet-induced EC activation, indicating that DKK1 promotes inflammation in atherosclerotic plaques and is an atherogenic factor.^9^ In a previous clinical study of patients with ACS, we found that DKK1 plasma levels were not only correlated with disease severity but also served as a prognostic predictor. Thus, DKK1 concentration may reflect the severity and stability of coronary atherosclerosis.^10^ Several studies have indicated that DKK1 plays an important role in atherosclerosis; however, the underlying mechanisms have yet to be elucidated. Furthermore, the knockout mouse is not an ideal model for DKK1 research in disease, because the homozygous DKK1 (-/-) mutation is lethal^11^. Therefore, in this investigation, we used a lentivirus to overexpress or silence DKK1 in ApoE-/- mice.

A previous study found a strong association between endoplasmic reticulum stress (ERS) markers, such as CCAAT/enhancer-binding protein-homologous protein (CHOP) and glucose-regulated protein 78 (GRP78), and the presence of atherosclerotic plaques in human coronary artery lesions, suggesting that ERS is involved in the development of plaque instability in humans.^12^ Disrupting the secretion of Wnt5a, a Wnt pathway agonist, has been shown to induce ERS in mammalian cells, indicating that a correlation exists between Wnt secretion and ERS.^13^ DKK1 is an important regulator of the Wnt pathway^8^, yet, its role in ERS-associated apoptosis in atherosclerosis remains unclear.

Based on these findings, we hypothesized that DKK1 promotes plaque formation and instability in part by stimulating EC apoptosis. To accomplish this, we investigated the effect of modulated DKK1 expression on atherosclerosis plaques in ApoE-/- mice and EC apoptosis; and explored the underlying mechanisms in endothelial cells using human umbilical vein endothelial cells (HUVECs).

**Results**

**DKK1 influenced the formation and vulnerability of aortic plaques and caused vascular endothelium dysfunction in ApoE-/- mice**

Intense GFP staining was observed in aortic plaques and carotid artery plaques (**Fig. 1b**). The results of Western blotting to reveal aorta-containing proteins, immunohistochemistry and analysis of plasma DKK1 further demonstrated that DKK1 protein expression was significantly lower in the shDKK1 group and higher in the DKK1 group than in the NS and GFP groups (**Fig. 1c-1f**), which established that overexpression and silencing vectors were effective.

Endomucin is a marker for endothelial cells^14^, and MOMA-2 is a marker for monocyte-macrophage^15^. Similar to the findings of a previous study, ^9^ atherosclerotic lesions in the aortic plaques in the ApoE-/- mice showed strong DKK1 expression in all endomucin-positive ECs and in small regions containing MOMA-2-positive macrophages (**Fig. 1g**). Colocalization immunofluorescence staining further confirmed that the majority of DKK1 was expressed in ECs other than smooth muscle cells and macrophages.

Vascular endothelium dysfunction plays an important role in atherosclerosis. When the mice were fed atherogenic chow for 4 weeks, the shDKK1 group showed recuperative acetylcholine-induced endothelium-dependent relaxation in the thoracic aorta ring after pre-contraction with norepinephrine, whereas the DKK1 group showed damaged endothelial relaxation (**Sul Fig.1**).

In mice fed atherogenic chow for 12 weeks, both the en-face lesion areas and the cross-section lesion areas measured in the aortic root were decreased in the shDKK1 group and increased in the DKK1 group compared with the areas in the NS and GFP groups (**Fig. 2a-c**).

In both aortic (**Fig. 2d**) and carotid (**Fig. 2h**) plaques, the relative content of VSMCs and collagen fibres were lower in the DKK1 group but higher in the shDKK1 group than in the NS GFP groups. Conversely, the relative contents of macrophages and lipids were higher in the DKK1 group but lower in the shDKK1 group than in the NS and the GFP groups. Accordingly, the plaque vulnerability index was higher in the DKK1 group but lower in the shDKK1 group. Moreover, fibrotic cap thickness was substantially lower in the DKK1 group and higher in the shDKK1 group than in the NS and the GFP groups (**Fig. 2d, 2g**). Although plaques were completely filled in most carotid arteries, except in the shDKK1 group, the carotid plaques were shown the similar trend (**Fig. 2h-2j**). Moreover, vascular outward remodelling was more significant in the DKK1 group than in the other groups (**Fig. 2k**). In mice fed atherogenic chow for 4 or 8 weeks, the shDKK1 group displayed substantially increased fibrous caps of plaque, while the DKK1 group showed increased plaque size and vulnerability (**Sul Fig. 2**). DKK1 can aggravate plaque vulnerability not only during the short-term feeding of atherogenic diet, but also in the long-term atherogenic diet. These results reveal that DKK1 augments plaque formation and instability in both aortic and carotid plaques and that silencing DKK1 attenuates these effects.

**DKK1 induced inflammatory factors and apoptosis in aortic atherosclerotic plaques in ApoE-/- mice**

Several studies showed that inflammation and apoptosis contribute to the instability of atherosclerotic plaques.^1^ The content of inflammatory factors (e.g., IL-6, IL-1β, MCP-1 and TNF-α) was lower in the shDKK1 group and higher in the DKK1 group (P<0.05, **Fig. 3a**).

Expression of the anti-apoptotic factor Bcl-2 was significantly upregulated in the shDKK1 group, while expression of the pro-apoptotic factor Bax and cleaved caspase-3 was significantly downregulated compared to the NS and GFP groups (P<0.05, **Fig. 3b**). The opposite results were found in the DKK1 group (P<0.05, **Fig. 3b**).

The percentage of TUNEL (+) cells was lower in the shDKK1 group and higher in the DKK1 group than in the NS and GFP groups (P<0.05, **Fig. 3c-3d**). The percentage of TUNEL (+) endomucin (+) cells showed a similar trend(P<0.05, **Fig. 3e**).Accordingly, the results from immunofluorescence analysis and TUNEL assay co-localization revealed that DKK1 promoted apoptosis in atherosclerotic plaques, mostly due to its effects on ECs.

**DKK1 expression was upregulated following ox-LDL treatment in a time- and concentration-dependent manner, and interference of DKK1 inhibited ox-LDL-induced apoptosis in HUVECs**

Oxidized low-density lipoprotein (ox-LDL) may play a preeminent function in atherosclerotic lesion formation^16^.So in vitro we used ox-LDL to mimic the stimulation to the endothelial cells during atherosclerosis. HUVECs treated with ox-LDL (150 µg/ml) for various lengths of time (0 h, 0.5 h, 1 h, 3 h, 6 h, or 12 h) exhibited time-dependent increases in DKK1 protein and mRNA levels as well as in DKK1 levels in the culture supernatant. DKK1 expression gradually increased at the mRNA level starting from 1h, at the protein level starting from 3h, and in the culture supernatant from 6h (P<0.05, **Fig. 4a-4c**). HUVECs treated for 6 h with various concentrations of ox-LDL (0 µg/ml, 25 µg/ml, 50 µg/ml, 100 µg/ml, 150 µg/ml, or 200 µg/ml) exhibited concentration-dependent increases in DKK1 mRNA and protein expression and in DKK1 levels in the culture supernatant. DKK1 expression was consistently significantly higher in cells treated with 150 µg/ml and 200 µg/ml ox-LDL than in cells treated with 0 µg/ml (P<0.05, **Fig. 4d-4f**). These results indicate that DKK1 expression increases in HUVECs in response to treatment with ox-LDL. Based on these results, we chose to treat cells with 150 µg/ml ox-LDL for 6 h to investigate how DKK1 affects EC function.

We first examined the effect of DKK1 on apoptosis in HUVECs treated with ox-LDL. To accomplish this, cells were transfected with either negative control siRNA (NC) or DKK1 siRNA. The DKK1 siRNA significantly reversed the effects of ox-LDL on the expression of cleaved caspase-3 and the protein ratio of Bcl-2 to Bax (P<0.05, **Fig. 4g**). Flow cytometry and TUNEL assay results consistently indicated that the percentage of cells undergoing apoptosis was significantly lower in the DKK1 siRNA group than in the NC group (P<0.05, **Fig. 4h-4k**).

**DKK1 induced apoptosis in HUVECs by activating ERS**

We next monitored changes in the expression of caspase-12, which is considered to be a marker of ERS-associated apoptosis in mice. Caspase-12 expression was reduced in the shDKK1 group and enhanced in the DKK1 group (P<0.05, **Fig. 3a**). To further investigate whether DKK1 induced apoptosis through ERS, we treated cells with 4-phenylbutyric acid (4-PBA), an ERS inhibitor.^17^Treatment with 4-PBA significantly attenuated the increase in cleaved caspase-3 expression and the decrease in the Bcl-2/Bax ratio observed in rDKK1-stimulated HUVECs (P<0.05, **Fig. 5a**) or lenti-DKK1-transfected HUVECs (P<0.05, **Sul Fig. 3a**). Notably, the TUNEL assay and flow cytometry results were consistent with those obtained by Western blotting (P<0.05, **Fig. 5b-5e**). In summary, we demonstrated that DKK1 induces apoptosis partly via the ERS pathway both in vivo and in vitro.

We also examined the effect of DKK1 on ERS. First, cells transfected with DKK1 siRNA were confirmed to have reduced DKK1 expression relative to cells transfected with NC siRNA. In contrast, cells were treated with ox-LDL or rDKK1 to induce the overexpression of DKK1. To determine whether ox-LDL activates ERS through DKK1, we monitored changes in the levels of eukaryotic initiation factor 2α (eif2α), CHOP, inositol-requiring enzyme 1 (IRE1), sliced X-box–binding protein 1 (XBP1s), transcription factor 6 (ATF6), and GRP78,^18^ which are considered markers of ERS.^19^ All of these markers showed significantly increased protein expression in HUVECs following treatment with ox-LDL or rDKK1 (P<0.05, **Sul** **Fig. 4a**). In addition, siRNA-mediated silencing of DKK1 reversed ox-LDL-induced ERS (P<0.05, **Sul** **Fig. 4b**). Based on these findings, we concluded that DKK1 may induce apoptosis in ox-LDL-treated HUVECs partly via ERS.

**DKK1 induced ERS-associated apoptosis through IRE1α and eif2α/CHOP**

We have monitored that DKK1 induced the level of eif2α, CHOP, IRE1, XBP1s, ATF6 and GRP78 in HUVECs. To identify the components playing a main role in DKK1-induced, ERS-associated apoptosis, we transfected HUVECs with NC siRNA, CHOP siRNA, or IRE1α siRNA prior to treatment with rDKK1or transfection with lenti-DKK1.^20^ After treatment with rDKK1 or transfection with lenti-DKK1, Bax and cleaved caspase-3 protein expression decreased, while Bcl-2 expression increased in cells transfected with CHOP siRNA or IRE1α siRNA compared to those transfected with NC siRNA (P<0.05, **Fig. 6a-6b,Sul Fig 3b-3c**). Flow cytometry and TUNEL assay results were consistent with those obtained by Western blotting (P<0.05, **Fig. 6c-6f**).

To further verify the role of eIF2α in DKK1-mediated effects, we also treated cells with salubrinal, which specifically inhibits ERS by preventing eIF2α dephosphorylation.^21-23^ Salubrinal significantly attenuated the increased cleaved caspase-3 expression and decreased Bcl-2/Bax expression in rDKK1-stimulated HUVECs (P<0.05, **Fig. 7a**) and lenti-DKK1 transfected HUVECs(P<0.05, **Sul Fig. 3d**). Moreover, the flow cytometry and TUNEL assay results were consistent with those obtained by Western blotting (P<0.05, **Fig. 7b-7e**). Generally, DKK1 induced apoptosis via IRE1α and eif2α/CHOP signaling, independent of other ERS transducers.

**DKK1 induced ERS-associated apoptosis through activation of the JNK pathway and inhibition of Wnt/β-catenin signaling**

We transfected cells with DKK1 siRNA to downregulate DKK1 and infected cells with lentivirus to overexpress DKK1. Western blotting revealed that DKK1 activated JNK, while knockdown of DKK1 led to a decrease in JNK phosphorylation (P<0.05, **Sul Fig.5a-5b**). To further verify the effect of JNK pathway on ER stress and apoptosis, we pretreated HUVECs with the JNK inhibitor-SP600125 significantly reversed the upward trend in GRP78 and CHOP protein expression observed in HUVECs transfected with lenti-DKK1 (P<0.05, **Fig. 8a**). These data indicate that DKK1 induces apoptosis and ERS in HUVECs by targeting and upregulating JNK. Furthermore, Bcl-2 expression increased significantly in lenti-DKK1-transfected HUVECs that were pretreated with SP600125, while Bax expression decreased.

IM-12 activates canonical Wnt signaling, ^24^ whereas FH535 acts as an inhibitor of canonical Wnt signaling. ^25^ To verify the effect of the canonical Wnt signaling pathway on apoptosis and ERS, we pre-treated HUVECs with IM-12 or FH535. HUVECs with pretreated IM-12 significantly reversed the upward trend in GRP78 and IRE1α protein expression in HUVECs transfected with lenti-DKK1 (P<0.05, **Fig. 8d**), while HUVECs with pretreated FH535 reverted the decrease in GRP78 and IRE1α protein expression in HUVECs transfected with DKK1 siRNA (P<0.05, **Fig. 8e**). However, the protein levels of JNK and CHOP were not changed in IM-12 or FH535.

**Discussion**

This is the first study to describe how DKK1 affects plaque formation and stability in atherosclerosis in ApoE-/- mice, which was accomplished by using lentivirus-mediated silencing and overexpression of the DKK1 gene.The following conclusions were generated: 1) The overexpression of DKK enlarged and destabilized atherosclerotic lesions and increased apoptosis, while inhibition of DKK1 expression hold the formation and vulnerability of atherosclerotic plaques in the whole progression of atherosclerosis; 2) treatment with ox-LDL induces DKK1 expression in HUVECs in a time- and concentration-dependent manner; 3) DKK1 induces ERS through IRE1α and eif2α/CHOP, leading to apoptosis; and 4) DKK1 activates ERS via both the JNK pathway and canonical Wnt signaling .

Our results show that DKK1 dose not influence the circulating levels of total cholesterol, LDL-C or blood glucose (**Sul Table 1**). Classical pathological studies have demonstrated that plaque components, inflammation factors and apoptosis play important roles in modulating the stability of atherosclerotic plaques.^26-28^ We found that DKK1 silencing reduced macrophage accumulation and increased VSMC numbers in plaques, while DKK1 overexpression augmented plaque vulnerability during the entire process of atherosclerosis.

# DKK1 is an antagonist of the Wnt signaling pathway.^29-32^ Ueland et al. found that DKK1 contributes to the activation of ECs by platelets.^9^ In a previous clinical study of patients with ACS, DKK1 plasma levels not only correlated with disease severity but also served as a prognostic predictor of disease, suggesting that DKK1 levels reflect coronary atherosclerosis stability.^10^ In two previous investigations conducted by our group, we found that treatment with ox-LDL promotes DKK1 expression in macrophages, resulting in inhibiting the accumulation of lipids^33^ and that oscillatory shear stress can induce DKK1 expression in ECs through PAR1/CREB.^34^ Both of these biomechanical and haemodynamic factors contribute to the development and destabilization of atherosclerosis. In earlier research, we used a partial carotid ligation model to imitate and induce disturbed flow and acute endothelial injury. ^34, 35^ Differ from it, we used constrictive silica collars to accelerate atherosclerotic lesion formation. ^36^ And we observed the lesion on both right carotid artery and aorta root 12 weeks’ atherogenic chow after collar surgery. Thus, in the [present](http://www.iciba.com/present) study, we demonstrated the negative effects of DKK1 on the formation and instability of atherosclerotic plaques.

In this study, we also observed that the expression of inflammatory factors (e.g., IL-6, IL-1β, TNF-α and MCP-1) decreased in conjunction with the downregulation of DKK1 and increased with upregulation of DKK1. Accumulation of inflammatory factors may induce monocyte recruitment and adhesion to the activated endothelial layer, thereby aggravating plaque instability.^6, 26-28^ Several studies have reported that the exogenous inhibition of DKK1 reduced IL-1β and TNF-α expression, significantly inhibited TNF-α expression in macrophage sand chondrocytes stimulated by lipopolysaccharide,^37^ attenuated angiogenesis,^38^ and decreased monocyte adhesion to HUVECs.^39^ DKK1 has also been shown to affect MMP-3 expression and influence collagen degradation in cartilage.^40^ Furthermore, DKK1 upregulation increases neovascularization.^41^ Thus, DKK1 may lead to plaque instability by augmenting inflammation, adhesion, collagen degradation and neovascularization.

In addition, we found that inhibition of DKK1 expression can attenuate cleaved caspase-3 expression as well as apoptosis. As a major regulator of the Wnt signaling pathway, DKK1 induces cellular apoptosis in many diseases.^29-32^ Overexpression of DKK1 sensitizes cells in brain tumours, ^42^ renal-cell carcinoma and thyroid cancers^43, 44^ to apoptosis. Weng et al. discovered that DKK1 expression is closely correlated with the expression of pro-apoptotic factors (e.g., Bad and caspase-3) in osteoarthritis and inhibition of DKK1 expression reduced caspase-3 cleavage and alleviated chondrocyte apoptosis by reducing Bax expression and increasing Bcl-2 expression.^40,45^ Cellular apoptosis is ubiquitous in vulnerable plaques; as such, recent studies on vulnerable plaques have mostly focused on inflammation and cellular apoptosis.

Multiple pathophysiological factors, both systemic and localized to the arterial walls, can disturb ER function in ECs, VSMCs, and macrophages during the initiation and progression of atherosclerosis.^46^ Expression of ERS activation markers has been observed in atherosclerotic lesions in humans and animals.^47^ Importantly, ERS-associated apoptosis is correlated with plaque instability and the clinical progression of atherosclerosis. CHOP is only robustly expressed in “vulnerable” plaques that show evidence of lesions and apoptosis.^47^ Moreover, Cominacini et al. revealed that persistent ERS is related to abnormal numbers of apoptotic cells in vulnerable plaques.^37^ Consistent with these findings, our results showed that inhibition of DKK1 expression remarkably reduced the expression of ERS-associated apoptotic markers (e.g., caspase-12) in ApoE-/- mice. Another study found that disrupting the secretion of human Wnt5a, a Wnt pathway agonist, induced ERS in mammalian cells, revealing a correlation between Wnt secretion and ERS.^13^ Our results indicate that DKK1 induces ERS and facilitates apoptosis in atherosclerosis.

In our study, ECs were found to be important sources of DKK1, as demonstrated by the co-localization of DKK1 with endomucin, an EC marker, in atherosclerotic lesions in ApoE-/- mice, which was in accord with the previous investigation.^9^ Interestingly, the observed areas of endomucin-positive ECs localization were almost equivalent to those that were stained positive for DKK1. Thus, even though DKK1 is expressed in different cell types，endothelial cells presumably played the most important roles other than smooth muscle cells and macrophages. While many different cells participate in ACS,^48^ apoptosis of ECs is a key event in the initiation of atherosclerotic plaque formation^47^ and the progression to advanced atherosclerosis, which is vulnerable to rupture.^3, 6, 7, 49-51^ We found that both SMCs and macrophages take up the GFP-labelled DKK1 from co-cultured ECs (**Sul Fig.6a**). In previous studies, recombinant DKK1 blocked the proliferation of VSMCs^52^ and inhibited foam cell formation in macrophages.^33^ Besides, inhibition of DKK1 expression can attenuate the inflammation of macrophages (**Sul Fig.6b-6c**).Thus, the effects of DKK1 in promoting the dysfunction and apoptosis of endothelial cells may be the “starting point” of DKK1’s effects on cross-talk with other cells in atherogenesis. ADMA^53^ and ET-1^54^ are markers of endothelial cell dysfunction. In our study, we also found that ADMA and ET-1 were positively correlated with DKK1 levels in plasma from healthy controls, unstable angina pectoris and acute myocardial infarction patients (p<0.05,S**ul Fig.7**).Therefore, we focused on ECs to experimentally determine the function of DKK1 in vitro.

Inhibition of DKK1 expression decreased ox-LDL-induced apoptosis and ERS in HUVECs. Prolonged and unresolvable ERS is known to induce apoptosis.^55, 56^ In particular, ERS promotes EC apoptosis via caspase-12 and the mitochondrial pathway.^57-58^ ERS has also been induced in ECs via pathological shear stress,^59^ hypoxia，^18, 57-58^ and increased GRP78, CHOP and caspase-12 expression. Here, for the first time, we showed that ox-LDL treatment activates ERS apoptosis via the JNK pathway, thereby promoting HUVECs apoptosis.

Numerous studies have shown that different cell types have specific ERS components; however, the effect of these variations on EC apoptosis is unclear. In 2005, Boyce et al. reported that salubrinal, a selective inhibitor of eif2α dephosphorylation, protects cells from ERS.[^3^](#_ENREF_2)^4^ In the present study, we used salubrinal, CHOP siRNA and IRE1α siRNA to inhibit various components associated with ERS and to investigate the role of eif2α, CHOP and IRE1α in ERS-associated apoptosis. Ultimately, we found that DKK1 induced ERS-associated apoptosis via IRE1α and eif2α/CHOP signaling. DKK1 is an antagonist of the canonical Wnt signaling pathway. We also found DKK1 can influence GRP78, IRE1α and apoptosis factors through Wnt/β-catenin signaling. However, Wnt/β-catenin signaling did not change the protein levels of JNK and CHOP.

In conclusion, inhibition of DKK1 expression effectively decrease plaque stability by attenuating ERS-mediated cellular apoptosis through intiating the JNK pathway and inhibiting Wnt/βcatenin. Moreover, the IRE1α and eif2α/CHOP pathways were found to participate in the activation of ERS. However, the mechanism underlying the augmentations of ERS by DKK1 remains to be determined. Notably, lentiviruses have low specificity in vivo. Therefore, in future experiments we will use EC-specific DKK1 KO mice and macrophage-specific DKK1 KO mice to determine the exact role of DKK1 in atherosclerosis through its action on various cell types. Although our data are preliminary, these findings might lead to new and promising methods for the treatment of atherosclerosis.

**Materials and Methods**

**Ethics statement**

All in vivo protocols involving animal care and experiments complied with the Guide for Care and Use of Laboratory Animals published by the United States National Institutes of Health (NIH Publication, 8th Edition, 2011) and the Animal Management Rules of the Chinese Ministry of Health (Document No. 55, 2001). All the in vivo experiments were approved by the Animal Care Committee of Shandong University. All the in vitro experimental protocols were approved by the Key Laboratory of Cardiovascular Remodeling and Function Research, Qilu hosipital, China. Human plasma samples were obtained from 72 patients of 45–75 years old patients in Qilu hospital. The research protocol was approved by the ethical committee of Qilu hospital, Shandong University.

**Atherosclerosis animal model protocol**

A total of 120 ApoE-/- mice (eight- week- old males) were purchased from the Peking University Animal Research Center (Beijing). All mice were fed atherogenic chow (i.e., a high-fat diet with 0.25% cholesterol and 15% cocoa butter) (**Fig. 1a**). The atherosclerotic model was created as previously described.^33^ We applied constrictive silica collars to the right carotid artery (RCA) to accelerate atherosclerotic lesion formation and investigated size, components, and vulnerability index in both aortic and carotid plaques. Pentobarbital sodium was used for anesthesia via [intraperitoneal](http://dict.youdao.com/w/intraperitoneal/#keyfrom=E2Ctranslation) [injection](http://dict.youdao.com/w/injection/#keyfrom=E2Ctranslation) (40 mg/kg) when placing the constrictive collars. The mice were randomly divided into four groups (n=30 each): a normal saline group (NS), an empty lentivirus group (GFP), a DKK1i lentivirus group (shDKK1), and a DKK1 lentivirus group (DKK1). Eight weeks after the surgery, a 200µl suspension (4*10^8^ TU DKK1i or DKK1 lentivirus per ml) was injected into each mouse through the tail vein. The mice were sacrificed 4 weeks post-transfection using pentobarbital sodium (50 mg/kg, i.p.) before exsanguination by perfusion via the abdominal aorta with PBS.

**Cell culture**

HUVECs were obtained from ScienCell Research Laboratories (Carlsbad, CA, USA) and cultivated in endothelial cell medium (ECM) (ScienCell, Carlsbad, CA) supplemented with 10% foetal bovine serum (FBS) and 1% penicillin/streptomycin at 37°C in 5% CO_2_. Cells from passages 4 to 8 were used for experiments. THP-1 cells obtained from the American Type Culture Collection(ATCC) .160 nM phorbol myristate acetate (PMA) was used overnight for THP-1 cell differentiation into macrophages.

**Lentiviral silencing and overexpression-vector construction**

To generate a lentivirus-mediated silencing vector, the lentivirus vector pGLV3/H1/GFP+Puro (pGLV3) was purchased from GeneChem (Shanghai, China), and a short-hairpin RNA sequence targeting DKK1 and or scrambled control RNA, was cloned into the vector. The following duplexes targeted murine DKK1: sense 5′-TCACCATCAAGCCAGCAAT-3′; antisense 5′- TCACCATCAAGCCAGCAAT-3′.

To achieve lentivirus-mediated DKK1 overexpression, the lentiviral vector LV5 was purchased from GenePharma Co., Ltd. (Shanghai, China), and the full-length coding sequence of either human or mouse DKK1 C-terminally tagged with green fluorescent protein (GFP) was cloned into the vector. A vector cloned with GFP alone was used as a negative control (NC).

**siRNA and RNA interference**

Upon reaching 40%-60% confluence, HUVECs were transfected with specific siRNA or negative control siRNA (GenePharma, Shanghai) (shown in **Supplemental Table 1**) using Lipofectamine 3000 ([Thermo Fisher Scientific](http://www.baidu.com/link?url=jwMMbDxFD6NqUcfiK7Rp7hnfkdoiRVIp2l167Aa-300V71UqLYhUBla5VDyn7L1UpwfGlQDVqBCeeVEf3O3Gv7zVNY_TpN8xm7S3-hmvltYnOA4Am5ENqOc-vmxqOSdQN6upLm5Wx_PTxSFJFe1XPa&wd=&eqid=b9e47a1d001ec73e0000000357158c5c), USA) in Opti-MEM (Gibco, Thermo Fisher Scientific, USA). After 6 h of transfection, the medium was replaced with complete ECM, and the cells were cultured for an additional 24 h. The transfected cells were treated with ox-LDL, recombinant DKK1 (rDKK1) or lenti-virus DKK1 at the designated concentrations and for the indicated times.

**Biochemical measurements**

The mice were fasted overnight. Blood samples were collected and centrifuged. Serum levels of total cholesterol, triglycerides, low-density lipoprotein cholesterol (LDL-C), high-density lipoprotein cholesterol and blood sugar were measured via enzymatic assay using an automatic biochemical analyser (Roche Cobas Integra 800, Basel, Switzerland).

**Histopathology and immunohistochemistry**

The whole aorta, the aortic root and the RCA were dissected, removed, fixed in 4% formaldehyde overnight at 4°C, embedded in OCT compound, prepared into 5-μm-thick sections. The cryosections were stained with haematoxylin and eosin for plaque morphology, oil red O for lipids, picrosirius red for collagen and Masson’s trichrome staining for fibrous cap. After blocking in 5% bovine serum albumin (BSA) in PBS, the sections were incubated with primary antibodies (shown in **Supplemental Table 2**) overnight at 4°C and then with an HRP Detection System (ZSGB-BIO). Detection was subsequently performed using DAB(3, 3′-diaminobenzidine) (ZSGB-BIO). Plaques stained with picrosirius red were viewed under polarized light. The areas of collagen, VSMCs, extracellular lipid deposits and macrophages were recorded as the percentage of positive area divided by the plaque area in 20 high-power fields (20*). The vulnerability index was calculated using the following formula: (macrophage staining%+lipid staining%)/(VSMC staining%+collagen staining%).^60^ Staining in the plaque was quantified using Image-Pro Plus 6.0 software (Media Cybernetics, USA) and a colour CCD video microscope (OLYMPUS, Japan).

**Immunofluorescence staining and microscopy**

The cryosections were blocked with 1% BSA and incubated with primary antibodies (shown in **Supplemental Table 2**) at 4 °C overnight. The sections were washed with PBS, and incubated with FITC- or TRITC-conjugated secondary antibodies. Nuclei were stained with 4′, 6-diamidino-2-phenylindole (DAPI; 1:2000, Roche, Germany) for 5 min. The samples were rinsed three times in PBS, and were examined under an epifluorescence microscope, and data were analysed using Image-Pro Plus 6.0 software (Media Cybernetics, USA).

**Aortic ring experiments in vitro**

Thirty ApoE-/- mice were injected NS, DKK1i or DKK1 lenti-virus respectively. The mice were sacrificed 2 weeks after transfection. The thoracic aortic rings 2-3 mm in depth that included the entire endothelium were cut and mounted in a 620M Multi myograph system (DMT, Denmark) containing 10 mL of Krebs bicarbonate solution maintained at 37 ºC. After [standardization](http://dict.youdao.com/w/standardization/#keyfrom=E2Ctranslation), [norepinephrine](http://dict.youdao.com/w/norepinephrine/#keyfrom=E2Ctranslation) (10^-5^M) was added to the bath to precontract the aortic ring, and stabilize for 5 min. Ach (10^-7^M, 10^-6^M, 10^-5^M) was [successively](http://dict.youdao.com/w/successively/#keyfrom=E2Ctranslation) added to the chamber to test for endothelial relaxation. The relaxation responses were calculated as a percentage of the [norepinephrine](http://dict.youdao.com/w/norepinephrine/#keyfrom=E2Ctranslation) pre-contraction. All the data were collected using Powerlab [software](http://dict.youdao.com/w/software/#keyfrom=E2Ctranslation) (AD Instruments, Australia).

**Western blot analysis**

HUVECs and tissue samples were lysed using RIPA buffer containing 1 mM phenylmethylsulfonyl fluoride (Invitrogen, Carlsbad, USA) and collected by centrifugation at 14,000 × rpm for 10 min. Equal amounts of proteins and pre-stained protein ladder (Thermo Fisher Scientific, USA) were separated on 10% SDS-PAGE gels, transferred to methanol-activated polyvinylidene fluoride membranes with a 0.45 µm pore size (Millipore, USA), and incubated with primary antibodies overnight at 4°C. The membranes were incubated with secondary antibodies (ProteinTech, USA) the next day for 1 h 20 min at room temperature. Bands were visualized using Immobilon ECL substrate (Millipore, USA), and blots were imaged with an LAS-4000 luminescent image analyser (Fujifilm, USA). Protein expression was quantified using Adobe Photoshop CS6 (Adobe Systems, USA), normalized to the β-actin expression in each sample, and expressed as a percentage of the control. The primary antibodies used in the experiments are shown in **Supplemental Table 2**.

**RNA extraction and quantitative real-time PCR**

Total RNA was extracted from HUVECs using TRIzol reagent (Ambion, Life Technologies, USA), and it was reverse-transcribed into cDNA using a PrimeScript™ RT Reagent Kit (TakaRa Biotechnology, Dalian, China). The cDNA (1 ng) was subjected to Q-PCR using SYBR Green (TakaRa Biotechnology, Dalian, China) for the relative quantification of mRNA expression. Quantification was accomplished using the 2-ΔΔCt method. β-actin was used to normalize mRNA levels. The reverse-transcription primer sequences used for the target genes are shown in **Supplemental Table 2**.

**ELISAs Kit**

The level of DKK1 in mice plasma were determined using a mouse DKK1 ELISA Kit (R&D, USA). The level of DKK1 in cell supernatant and human plasma was assessed using a human DKK1 ELISA Kit (R&D, USA). Other protein levels were determined using the following ELISA kits: ADMA ELISA Kit (BlueGene Biotech, China), ET-1 ELISA Kit (BlueGene Biotech, China). All kits were used according to the manufacturer’s protocol. The analysis was completed at a wave-length of 450 nm (with reference of 570 nm) in an ELISA plate reader.

**Flow cytometry**

Cell apoptosis was analysed using an Annexin V PE/7-Amino-Actinomycin (7-AAD) Apoptosis Detection Kit (BD Pharmingen, USA) according to the manufacturer’s protocol. The following groups were used to set up compensation and to define quadrants: unstained control cells, cells stained with PE Annexin V (no 7-AAD), and cells stained with 7-AAD (no PE Annexin V). Apoptotic cells were examined using a flow cytometer (Becton-Dickinson, USA) within 1 h, and the percentage of early apoptotic cells (upper-right quadrant) was measured using FlowJo software (China).

**In situ detection of apoptotic cells**

Apoptotic cells and apoptotic ECs in aortic root cryosections were determined using an In Situ Apoptosis Fluorescein Detection Kit (Millipore, Billerica, MA, USA).

**Statistical analysis**

Data were analysed using SPSS v16.0 (SPSS Inc., Chicago, IL). Data are presented as the mean ± S.D. of at least three independent experiments. Normality of variables distribution was tested by the Kolmogorov-Smirnov test. Comparisons were analysed using Student’s t test or one-way ANOVA followed by Bonferroni post hoc test. P<0.05 was considered statistically significant.

**Acknowledgements:** This study was supported by grants from the National Natural Science Foundation of China (No. 81270404, 81470559).

**Conflict of Interest:** None declared

**References**  **Figure Legends:**

**Figure 1: Efficiency of lentivirus transfection in ApoE-/- mice.**

**(a).** Flow charts showing the experimental protocol used in the *in vivo* studies. **(b).** Fluorescence images of the aortic root and carotid plaques two weeks after lentivirus transfection in the lenti-GFP group. **(c-d).** DKK1 protein expression in the aortic root in four groups of mice as determined by Western blotting. n=6. **(e).** Areas stained positive for DKK1 in four groups of mice as determined by immunohistochemical staining in the aortic root and carotid plaques. n=6. **(f).** Plasma levels of DKK1 in four groups of mice. (n=11). **(g).** Co-localization of DKK1 (red) and endomucin (green) or MOMA-2 (green) expression in aortic plaques in the NS group. DAPI (blue) indicates nuclei. Data are shown as the mean±S.D.,* P<0.05 vs. NS; # P<0.05 vs. GFP.

**Figure 2: Influence of DKK1 on plaque formation and stability in ApoE-/- mice.**

**(a).** En face Oil Red O staining of aortas and cross-sectional aortic root lesions with H&E staining in four groups of mice (NS, GFP, shDKK1, and DKK1). **(b).** Quantitative analysis of en face aortic lesions expressed as percentage lesion area relative to total aorta area. n=6. **(c).** Quantitative analysis of cross-sectional plaque areas in aortic roots. n=6. **(d-e, h-i).** Representative immunohistochemical staining and quantification of plaque content in aortic plaques and carotid artery plaques**.** n=6. **(f. j).** Quantitative analysis of plaque vulnerability indices of the aortic plaques and carotid artery plaques. **(g).** Quantitative analysis of plaque fibrotic cap thickness of aortic plaques. n=5. **(k).** Quantitative analysis of plaque area and total lumen area in the carotid artery. n=6.Data are shown as the mean±S.D.* P<0.05 vs. NS; # P<0.05 vs. GFP.

**Figure 3: Effects of DKK1 on inflammatory factors and plaque apoptosis in ApoE-/-**

**(a).** Representative immunohistochemical staining and quantification of plaque content of inflammatory factors (IL-6, IL-1β, MCP-1,TNF-α). n=6. **(b).** Representative immunohistochemical stained images and quantification of Bax, Bcl-2, cleaved caspase-3 and caspase-12 levels in atherosclerotic plaques of ApoE-/- mice. n=6. **(c).** Co-localization and quantitative analysis of TUNEL staining (green) and endomucin-positive areas (red) to measure cellular apoptosis in aortic plaque. DAPI (blue) indicates nuclei. n=3. Data are shown as the mean ±S.D.,* P<0.05 vs. NS; # P<0.05 vs. GFP.

**Figure 4: Time- and dose-dependent effects of ox-LDL treatment on the expression of DKK1 and attenuation of ox-LDL-induced apoptosis in HUVECs following DKK1 knockdown. (a-c):** Quantification of DKK1 expression in HUVECs treated with ox-LDL (150 µg/ml) for various lengths of time: **(a)** DKK1 mRNA levels, n=3. **(b)** protein expression levels, n=6. **(c)** Levels in culture supernatant by ELISA, n=3. **(d-f):** Quantification of DKK1 expression in HUVECs treated for 6 h with various concentrations of ox-LDL: **(d)** DKK1 mRNA, n=3. **(e)** Protein expression levels, n=6. **(f)** Levels in culture supernatant by ELISA, n=3. **(g-k):** HUVECs were transiently transfected with negative control (NC) and DKK1 siRNA (si-DKK1) for 24 h and then treated with 150 µg/ml ox-LDL for 6 h. **(g)** Western blotting to quantify Bax, Bcl-2, cleaved caspase-3 and DKK1 protein levels. n=6. **(h, j)** Flow cytometric analysis to quantify early apoptotic cells (i.e., Annexin V-positive and 7AAD-negative cells, lower-right quadrant). n=3. **(i, k)** HUVECs with stained nuclei (green) were considered TUNEL-positive (red arrows). The percentage of TUNEL-positive cells was calculated and quantified. n=6. Data are shown as the mean ±S.D.*P<0.05 vs. the untreated group or NC; # P<0.05 vs. NC+ ox-LDL.

**Figure5: DKK1 promotes apoptosis via endoplasmic reticulum stress in HUVECs.** Cells were pretreated with PBS or 4-PBA (3 mM or 5 mM) for 1 h before and during rDKK1 (300 ng/ml) treatment. **(a).** Western blotting to quantify cleaved caspase-3, Bcl-2, and Bax protein expression. n=6. **(b, d).** HUVECs with stained nuclei (green) were considered TUNEL-positive (arrows). The percentage of TUNEL-positive cells was calculated and quantified. n=3. **(c, e).** Flow cytometric analysis for quantification of early apoptotic cells (i.e., Annexin V-positive and 7AAD-negative cells, lower-right quadrant). n=3. Data are shown as the mean±S.D. *P<0.05 vs.control; # P<0.05 vs. only rDKK1 treatment.

Figure 6: **DKK1 activates CHOP and IRE1α signaling during ERS to induce apoptosis**

HUVECs were transiently transfected with negative control (NC) siRNA, CHOP siRNA (si-CHOP) or IRE1α siRNA (si-IRE1α) for 24 h and then treated with rDKK1 (300 ng/ml) for 6 h. **(a, b).**Western blotting for quantification of the protein levels of cleaved caspase-3, Bcl-2 and Bax. n=6. **(c, e).** HUVECs with stained nuclei (green) were considered TUNEL-positive (arrows). The percentage of TUNEL-positive cells was calculated and quantified. n=3. **(d, f).** Flow cytometric analysis for quantification of early apoptotic cells (i.e., Annexin V-positive and 7AAD-negative cells, lower-right quadrant). n=3. Data are shown as the mean ±S.D.*P<0.05 vs.NC or control; # P<0.05 vs. only rDKK1 treatment.

Figure 7: **DKK1 activates eif2α during ERS to induce apoptosis**

Cells were pretreated with DMSO or salubrinal (50 µM) for 1 h before rDKK1 (300 ng/ml) treatment. **(a).**Western blotting for quantification of the protein levels of cleaved caspase-3, Bcl-2 and Bax. n=6. **(b, d).** HUVECs with stained nuclei (green) were considered TUNEL-positive (arrows). The percentage of TUNEL-positive cells was calculated and quantified. n=3. **(c, e).** Flow cytometric analysis for the quantification of early apoptotic cells (i.e., Annexin V-positive and 7-AAD-negative cells, lower-right quadrant). n=3. Data are shown as the mean ±S.D.*P<0.05 vs.NC or control; # P<0.05 vs. only rDKK1 treatment.

**Figure 8: DKK1 induces apoptosis by promoting ERS via activation of the JNK pathway and inhibition of canonical Wnt signaling. (a-c).** Cells were pretreated with DMSO or SP600125 (10 µM) for 1 h before transfection with lenti-DKK1. Western blotting for quantification of cleaved caspase-3, Bcl-2, Bax, CHOP and GRP78 protein levels. n=6. Data are shown as the mean ±S.D.*P<0.05 vs. DMSO; # P<0.05 vs. rDKK1 treatment only. **(d, f, h).** Cells were pretreated with DMSO or IM-12 (3 µM) for 1 h before and during transfection with lenti-DKK1. Western blotting for quantification of cleaved caspase-3, Bcl-2, Bax, p-JNK, CHOP, IRE1α and GRP78 protein levels. n=6. Data are shown as the mean ±S.D.*P<0.05 vs. DMSO; # P<0.05 vs. lenti-DKK1 transfection. **(e, g, i).** Cells were pretreated with DMSO or FH535 (30 µM) for 1 h before and during transfection with DKK1 siRNA. Western blotting for quantification of cleaved caspase-3, Bcl-2, Bax, p-JNK, CHOP, IRE1α and GRP78 protein levels. n=6. Data are shown as the mean ±S.D. *P<0.05 vs. DMSO; # P<0.05 vs. DKK1 shRNA transfection. (**j**) Proposed model of the DKK1 signaling pathway responsible for cell apoptosis.

**Supplemental** **Figure 1**: **Concentration-response curves for acetylcholine in aortic ring segments from mice transfected with lenti-DKK1 or lenti-DKK1i for 2 weeks, compared with rings from control mice.** n=8. Mean± S.D., *P<0.05, vs control. NE: norepinephrine.

**Supplemental** **Figure2: Influence of DKK1 on early atherogenesis in ApoE-/- mice.**

**(a).** Flow charts showing the experimental protocol used in the *in vivo* studies. **(b-c).** Quantitative analysis of cross-sectional plaque area in aortic roots. n=6. **(b, d).** Quantitative analysis of plaque fibrotic cap thickness in aortic plaques. n=4. **(b, e).** Quantitative analysis of plaque vulnerability index for aortic plaques and carotid artery plaques. n=4. **(b, f).**Representative immunohistochemical staining and quantification of plaque content in aortic plaques from animals fed for 4 weeks with atherogenic chow**.** n=6. **(b, g).** Representative immunohistochemical staining and quantification of plaque content in aortic plaques from animals fed for with 8 weeks with atherogenic chow**.** n=6. Data are shown as the mean ±S.D.* P<0.05 vs. NS; # P<0.05 vs. GFP.

**Supplemental** **Figure 3: DKK1 promotes apoptosis via endoplasmic reticulum stress in HUVECs.**

**(a).** Cells were pretreated with PBS or 4-PBA (3 mM or 5 mM) for 1 h before transfected with lenti-DKK1. Western blotting to quantify cleaved caspase-3, Bcl-2, and Bax protein expression. **(b-c)** HUVECs were co-transfected with lenti-DKK1 and negative control (NC) siRNA, CHOP siRNA (si-CHOP) or IRE1α siRNA (si-IRE1α) for 48 h. Western blotting for quantification of the protein levels of cleaved caspase-3, Bcl-2 and Bax. **(d)** Cells were pretreated with DMSO or salubrinal (50 µM) for 1 h before DKK1 transfection. Western blotting for quantification of the protein levels of cleaved caspase-3, Bcl-2 and Bax. Data are shown as the mean ±S.D. *P<0.05 vs.control; # P<0.05 vs. only lenti-DKK1 group.

**Supplemental** **Figure 4: Effects of DKK1 on ERS.**

Western blotting for quantify of the protein levels of p-eif2α, CHOP,p- IRE1α, XBP1s, ATF6, and GRP78. **(a).** HUVECs were treated with ox-LDL (150 µg/ml) or rDKK1 (300 ng/ml) for 6 h. n=6. Data are shown as the mean ±S.D.*P<0.05 vs. control. **(b).** HUVECs were transiently transfected with negative control (NC) or DKK1 siRNA (si-DKK1) for 24 h, and then treated with ox-LDL for 6 h. n=6.Data are shown as the mean ±S.D.*P<0.05 vs. NC, # P<0.05 vs. NC+ ox-LDL.

**Supplemental** Figure 5: **Effects of DKK1 on** the JNK pathway

**(a).** Western blotting for quantification of JNK phosphorylation after transfection with lentivirus-DKK1 for 24 h. n=3. * P<0.05 vs. CON; # P<0.05 vs.MOCK. **(b).** Western blotting for quantification of JNK phosphorylation after transfection with negative control (NC) or DKK1 siRNA (si-DKK1) for 24 h. n=3. Data are shown as the mean ±S.D.,* P<0.05 vs. control; # P<0.05 vs. NC.

**Supplemental** **Figure 6: Autocrine and paracrine DKK1 from HUVECs and DKK1’s effects on macrophages’ inflammation**

**(a)** HUVECs were transfection with GFP labelled lenti-DKK1 for 24 h. HUVECs, SMCs, macrophage were co-cultured with GFP-labelled-DKK1 transfected HUVECs or control. GFP (green) expression in the basement cells. Western blotting for confirm the presence of GFP-DKK1 in the basement cells. **(b)** Attenuation of ox-LDL-induced inflammation in macrophages following transfected with DKK1 siRNA. n=3. Data are shown as the mean ±S.D.,*P<0.05 vs. the untreated group or NC; # P<0.05 vs. NC+ ox-LDL.

**Supplemental Figure 7：Correlations between plasma DKK-1 concentration and asymmetric dimethylarginine (ADMA), endothelin (ET-1) concentration.** CON: agematched healthy control; UAP: unstable angina pectoris; AMI: acute myocardial infarction. n=24. Data are shown as the mean ±S.D., n.s, insignificance, ** P<0.01 vs. CON.
